# Antimicrobial Efficacy of Contact Lens Solutions Assessed by ISO Standards

**DOI:** 10.3390/microorganisms9102173

**Published:** 2021-10-19

**Authors:** Cindy McAnally, Rhonda Walters, Allison Campolo, Valerie Harris, Jamie King, Megan Thomas, Manal M Gabriel, Paul Shannon, Monica Crary

**Affiliations:** Alcon Research, LLC, Fort Worth, TX 76134, USA; rhonda.walters@alcon.com (R.W.); allison.campolo@alcon.com (A.C.); valerie-1.harris@alcon.com (V.H.); jamie.king@alcon.com (J.K.); megan.thomas@alcon.com (M.T.); manal.gabriel@alcon.com (M.M.G.); stephen.shannon@alcon.com (P.S.); monica.crary@alcon.com (M.C.)

**Keywords:** microbial keratitis, serratia, fusarium, contact lens, multipurpose disinfecting solution

## Abstract

Microbial keratitis (MK) is an eye infection caused by opportunistic bacteria or fungi, which may lead to sight-threatening corneal ulcers. These microorganisms can be introduced to the eye via improper contact lens usage or hygiene, or ineffective multipurpose solutions (MPSs) to disinfect daily wear contact lenses. Thus, the patient’s choice and use of these MPSs is a known risk factor for the development of MK. It is then critical to determine the efficacy of popular MPSs against ubiquitous ocular microorganisms. Therefore, we compare the efficacy of nine major MPSs on the global market against four different microorganism species, and with four different common contact lenses. In accordance with International Standards Organization protocol 14729 and 18259, the microorganisms were inoculated into each MPS with and without contact lenses, and held for the manufacturer’s disinfection time, 24 h, and 7 days after challenge with *Serratia marcescens* or *Fusarium* spp. Plates were incubated for 2–7 days and plate counts were conducted to determine the number of surviving microorganisms. The majority of MPSs demonstrated significantly higher disinfection efficacies without contact lenses. Broadly, among the microorganisms tested, the OPTI-FREE products (Puremoist, Express, and Replenish) maintained the highest disinfection efficacies at the manufacturer’s stated disinfection time when paired with any contact lens, compared with other MPSs. These were followed closely by RevitaLens and renu Advanced. MPSs containing dual biocides polyquaternium-1 and myristamidopropyl dimethylamine possessed the highest disinfection efficacy against multiple ocular pathogens.

## 1. Introduction

Microbial keratitis (MK), an infection resulting in corneal ulceration, is a sight-threatening complication affecting over 30,000 people in the United States per year [1]. Although the opportunistic microorganisms responsible for these ocular infections are ubiquitous and usually considered harmless to otherwise healthy individuals, the largest risk factors for contact lens wearers developing MK are poor contact lens and storage case hygiene (topping off the disinfecting multipurpose solution (MPS), inadequate cleaning and disinfection of lenses, infrequent case replacement) or less robust MPSs [1]. Ineffective MPSs have been directly linked to major MK outbreaks in the United States and around the world. Therefore, the differences in how well the biocides within each MPS are able to disinfect any particular contact lens are critical [1,2,3,4,5,6].

Notably, within these MK outbreaks, *Fusarium* is a widely distributed genus of filamentous fungi that usually poses little danger to healthy individuals. However, *Fusarium* species have been found to be the causative microorganisms in a number of MK cases [7], as this opportunistic microorganism can be introduced to the eye via improper contact lens hygiene habits or ineffective biocides [8]. In particular, past *Fusarium* keratitis outbreaks in the United States, Hong Kong, and Singapore have been found to specifically be the result of an ineffective MPS, renu with MoistureLoc [2,4]. Further, although fungal infections in general always pose a substantial challenge in treatment, because these microorganisms are biologically similar to human cells, the *Fusarium solani* species complex [9] responsible for these ocular infections is considered highly resistant to antifungal strategies [10]. Thus, it becomes that much more imperative to reduce *Fusarium* contamination. In addition, it is important to protect against the common prokaryotic microorganisms responsible for MK, such as *Serratia*, which is one of the most frequently isolated bacteria in MK cases [11,12]. Critically, these represent some of the most challenging microorganisms when testing disinfection efficacy of contact lens care products, and are thus suitable for identifying the most robust products.

The MPSs that are commonly available and marketed to disinfect contact lenses contain a myriad of biocides that have been previously shown to be effective against common ocular pathogens [13]. To note, readily available ophthalmic solutions have also been shown to be effective against SARS-CoV-2, which can infect a host via the eye [14]. Currently, there is no evidence of antiviral activity of any MPS against SARS-CoV-2, although a study has recently shown that the rub and rinse instructions for use were effective at reducing the viral load on contact lenses [15]. However, the type and quantity of each biocide within a particular MPS can produce stark differences in disinfection capabilities overall [13]. MPSs are evaluated for antimicrobial activity by two International Standards Organization (ISO) methods: ISO 14729 and ISO 18259 [16,17]. ISO 14729 requires a three log reduction for bacteria and a one log reduction for yeast and mold at disinfection time (DT) to meet primary criteria. If an MPS cannot meet this requirement, a manufacturer may demonstrate that the MPS meets secondary criteria, which requires evidence of disinfection when utilizing the directions for use. Further, as previous investigations have indicated that the presence of contact lenses can reduce the antimicrobial efficacy of MPSs [13], ISO 18259 was developed for manufacturers to assess the impact of contact lenses and contact lens cases on the antimicrobial efficacy of their solutions. It is important that MPSs are tested both with and without lenses present in order to fully understand real-world applications and efficacies of any MPS. These tests should additionally be undertaken in the manufacturer-provided contact lens cases, as biocide uptake by contact lens cases can introduce significant reductions to the presence and activity of MPSs [13,18]. Although we have previously shown that commonly available MPSs demonstrate strong biocidal efficacy against ocular pathogens such as *Staphylococcus aureus*, *Pseudomonas aeruginosa*, and *Candida albicans* [13], previous investigations have yet to fully examine the more novel contact lenses available after challenge with resistant infectious microorganisms, such as the *Fusarium* and *Serratia* species. Further, the most recent additions to the contact lens product market have not been fully scrutinized in this way.

Thus, the present study aims to investigate the ability of nine different preserved MPSs containing a range of biocides to maintain sufficient antimicrobial activity in the presence of four different types of contact lenses against common ocular microorganisms. Further, we compare the results obtained with ISO 14729 and ISO 18259 to assess intrinsic antimicrobial efficacy versus simulated patient use disinfection for these MPS systems.

## 2. Materials and Methods

### 2.1. Preparation of Microorganism Suspensions

The intrinsic antimicrobial efficacy was determined by using ISO 14729 (“Ophthalmic optics—Contact lens care products–Microbiological requirements and test methods for products and regimens for hygienic management of contact lenses”) [16] test methods and criteria. In addition, antimicrobial efficacy endpoint methodology compatibility (AEEMC) was performed in accordance with the ISO 18259 (“Ophthalmic optics–-Contact lens care products–Method to assess contact lens care products with contact lenses in a lens case, challenged with bacterial and fungal organisms”) [17] protocol methodology. Commercially sourced contact lenses and MPSs used in this study, and their active biocide concentrations are listed in Table 1. Both ISO 14729 and ISO 18259 antimicrobial efficacy testing were performed for the indicator microorganisms at disinfection time, with additional time points assessed for ISO 18259. Microorganisms were acquired from the American Type Culture Collection (ATCC; Manassas, VA, USA) and the Alcon Laboratories Microbial Collection (AMC; Fort Worth, TX, USA). Bacterial cultures (*Serratia marcescens,* ATCC 13880) were transferred to soybean casein digest agar (SCDA) and were incubated for 18–24 h at 30–35 °C. Cells were harvested using 0.9% saline with 0.1% peptone. Mold cultures (*Fusarium keratoplasticum* [formerly identified as *Fusarium solani*], ATCC 36031; *Fusarium chlamydosporum*, AMC 5663; and a clinical keratitis isolate of *Fusarium,* AMC 1620) were transferred to potato dextrose agar and incubated for 10–14 days at 20–25 °C. Spores were harvested using Dulbecco’s phosphate buffered saline with 0.05% polysorbate 80 and filtered through glass wool. Following these steps, the microorganism suspensions were adjusted to concentrations of approximately 2.0 × 10^7^ to 2.0 × 10^8^ colony forming units (CFU) per mL, and resuspended in a 10% organic soil suspension containing heat-killed *Saccharomyces cerevisiae* cells (ATCC 9763; 10^7^ to 10^8^ CFU/mL) and heat-inactivated fetal bovine serum (VWR, Radnor, PA, USA).

### 2.2. Stand-Alone Inoculation with Microorganisms

ISO 14729 was performed by inoculating 1.0 × 10^5^ to 1.0 × 10^6^ CFU/mL of the tested microorganisms into test tubes containing 10 mL of the required MPS. Test tubes were made of compatible materials based on the biocides within the MPS. Test controls (diluents) were prepared in the same manner. Test samples and controls were evaluated to determine the number of surviving microorganisms at the recommended disinfection time.

### 2.3. Contact Lens Inoculation with Microorganisms

For ISO 18259, contact lenses were removed aseptically from the blister package and soaked in phosphate buffered saline for 18 h. The lenses were blotted on sterile gauze to remove excess solution, and placed in the matching manufacturer’s contact lens case with the concave side up. Lenses were then inoculated to contain a final count of 1.0 × 10^5^ to 1.0 × 10^6^ CFU/mL of the tested microorganism. Following a contact time of 3–10 min, the required MPS was added to the cases (to the fill line) and the cases were closed, giving special attention to not contaminate the cap. Closed cases were stored at 20–25 °C. Test controls (MPSs without lenses) were prepared in the same manner. Separate lenses/cases were prepared for each specific sampling time to avoid opening and closing, or re-entering cases before their final endpoint. Test samples and controls were evaluated to determine the number of surviving microorganisms at the recommended disinfection time as well as additional time points at 24 h and 7 days. The lens cases were vortexed vigorously for 30 s prior to sampling. Lenses were removed and discarded per protocol.

### 2.4. Microorganism Recovery

To recover surviving microorganisms for both ISO standards, aliquots (1 mL) of the solution or lens/solution combinations and their controls were transferred to test tubes containing 9 mL Dey–Engley neutralizing broth (DE broth, Difco). Serial 1:10 dilutions were conducted using additional test tubes containing DE broth. Appropriate neutralization times were validated prior to testing and observed. DE broth was shown to be effective at neutralizing antimicrobial agents contained in the test solutions. The recovery of microorganisms from the neutralizing broth with products was within 50% of the recovery of microorganisms from the control tube (no product) for all test microorganisms. 

### 2.5. Microorganism Quantification

Dilutions were then plated to quantify the CFU/mL. Bacterial and fungal pour plates were prepared with SCDA containing 0.07% lecithin and 0.5% polysorbate 80. Bacterial plates were incubated for 2–5 days at 30–35 °C and mold plates were incubated for 5–7 days at 20–25 °C. Following the incubation periods, plate counts were conducted and the CFU/mL was calculated based on the average from duplicate plates. 

### 2.6. Statistical Analysis

Quantifications were analyzed via one-way ANOVA with either a post-hoc Dunnett’s test (when comparing MPS challenges to no-lens challenges) or Tukey’s test (when comparing product families against each other), with *p* < 0.05 being used for significance.

## 3. Results

**Figure 1:**
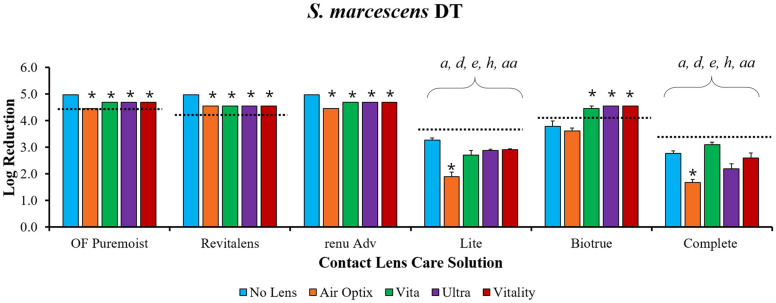
Multipurpose solutions (MPSs) and contact lenses challenged with *Serratia marcescens* ATCC 13880 at the manufacturer’s disinfection time (DT). Disinfection efficacy is stated in mean ± standard error log reduction compared to inoculum control. The dashed line represents ISO 14729 antimicrobial efficacy testing. * *p* < 0.05 vs. no-lens challenge at the same time point and within the same MPS. Statistical differences among product families: *a p* < 0.05 vs. OPTI-FREE (OF) Puremoist with Air Optix HG, *d p* < 0.05 vs. RevitaLens with Vita, *e p* < 0.05 vs. renu Advanced with Ultra, *h p* < 0.05 vs. Biotrue with Ultra; *aa p* < 0.05 vs. OPTI-FREE Puremoist with other manufacturer lens. *n* = 3/group.

**Figure 2:**
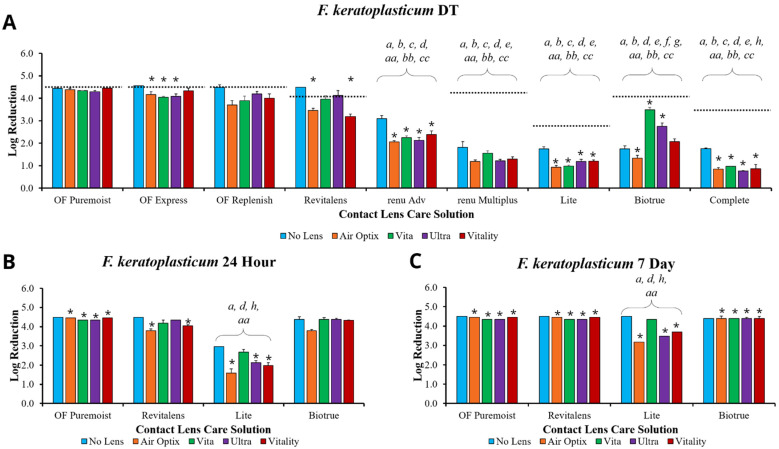
Multipurpose solutions (MPSs) and contact lenses challenged with *Fusarium keratoplasticum* ATCC 36031 at (**A**) the manufacturer’s disinfection time (DT), (**B**) 24 h, and (**C**) 7 days. Disinfection efficacy is stated in mean ± standard error log reduction compared to inoculum control. The dashed line represents ISO 14729 antimicrobial efficacy testing. * *p* < 0.05 vs. no-lens challenge at the same time point and within the same MPS. Statistical differences among product families: *a p* < 0.05 vs. OPTI-FREE (OF) Puremoist with Air Optix HG, *b p* < 0.05 vs. OPTI-FREE Express with Air Optix HG, *c p* < 0.05 vs. OPTI-FREE Replenish with Air Optix HG, *d p* < 0.05 vs. RevitaLens with Vita, *e p* < 0.05 vs. renu Advanced with Ultra, *f p* < 0.05 vs. renu MultiPlus with Ultra, *g p* < 0.05 vs. Lite with Vitality, *h p* < 0.05 vs. Biotrue with Ultra; *aa p* < 0.05 vs. OPTI-FREE Puremoist with other manufacturer lens, *bb p* < 0.05 vs. OPTI-FREE Express with other manufacturer lens, *cc p* < 0.05 vs. OPTI-FREE Replenish with other manufacturer lens. *n* = 3/group.

**Figure 3:**
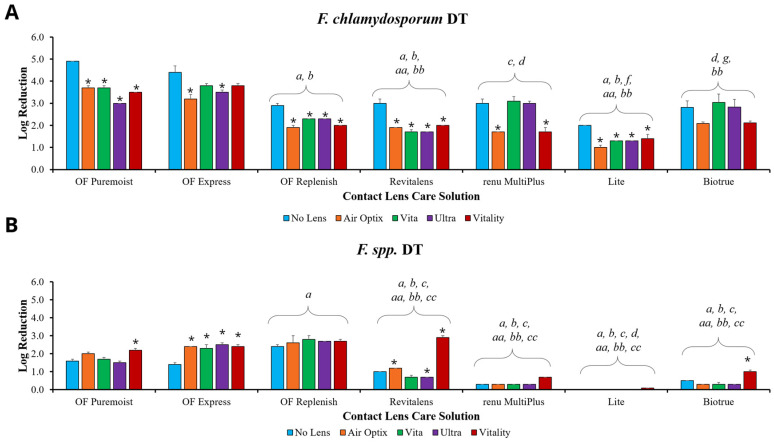
Multipurpose solutions (MPSs) and contact lenses challenged with (**A**) *Fusarium chlamydosporum* AMC 5663 and (**B**) clinical keratitis isolate *Fusarium* AMC 1620 at the manufacturer’s disinfection time (DT). Disinfection efficacy is stated in mean ± standard error log reduction compared to inoculum control. * *p* < 0.05 vs. no-lens challenge at the same time point and within the same MPS. Statistical differences among product families: *a p* < 0.05 vs. OPTI-FREE (OF) Puremoist with Air Optix HG, *b p* < 0.05 vs. OPTI-FREE Express with Air Optix HG, *c p* < 0.05 vs. OPTI-FREE Replenish with Air Optix HG, *d p* < 0.05 vs. RevitaLens with Vita, *f p* < 0.05 vs. renu MultiPlus with Ultra, *g p* < 0.05 vs. Lite with Vitality; *aa p* < 0.05 vs. OPTI-FREE Puremoist with other manufacturer lens, *bb p* < 0.05 vs. OPTI-FREE Express with other manufacturer lens, *cc p* < 0.05 vs. OPTI-FREE Replenish with other manufacturer lens. *n* = 3/group.

To determine the disinfection efficacy of each MPS, solutions were challenged with microorganisms, with and without contact lenses (Figure 1, Figure 2 and Figure 3). The disinfection efficacy of each MPS was determined by calculating the log reduction of microbial growth compared to the paired inoculum control (see Table S1, Supplemental Digital Content, which describes the CFU/mL of the inoculum control of each organism). Although ISO 14729 requires five microorganisms for testing, this study only presents microorganisms where clear product differentiation was demonstrated. Thus, data for *Pseudomonas aeruginosa* ATCC 9027, *Staphylococcus aureus* ATCC 6538, and *Candida albicans* ATCC 10231 are not presented here, but have been shown in previous studies [13].

When MPSs without contact lenses were challenged with *Serratia marcescens* (Figure 1), OPTI-FREE Puremoist, RevitaLens, and renu Advanced demonstrated a five log reduction after soaking for the manufacturer’s recommended disinfection time. When disinfection time was increased to 24 h, Biotrue also achieved a five log reduction, and Lite and Complete achieved 4.8 and 4.6 log reductions, respectively. Complete achieved 4.8 and 4.6 log reductions, respectively. Disinfection efficacy was significantly reduced when MPS challenges included contact lenses (*p* < 0.05). OPTI-FREE Puremoist, RevitaLens, and renu Advanced maintained the disinfection efficacy from the manufacturer’s disinfection time through 24 h for both no-lens challenges and when lenses were included (see Figure S1, Supplemental Digital Content, which illustrates the quantification of *Serratia marcescens* log reduction at 24 h). When used for the manufacturer’s disinfection time, Biotrue was the only MPS to maintain greater disinfection efficacy when used with lenses than without. Product families (i.e., the paired MPS and contact lens from a single company, Table 1) were also compared. When following the manufacturer’s disinfection time, Lite (paired with Vitality) and Complete (paired with Vita) both maintained significantly lower log reductions than OPTI-FREE Puremoist (with Air Optix HG), RevitaLens (with Vita), renu Advanced (with Ultra), or Biotrue (with Ultra). Similarly, the pairing of OPTI-FREE Puremoist with other manufacturer lenses was compared to the manufacturer pairing of lenses and solutions (*p* < 0.05). Lite/Vitality was significantly less effective than OPTI-FREE Puremoist/Vitality, and Complete/Vita was significantly less effective than OPTI-FREE Puremoist/Vita (*p* < 0.05). 

As *Fusarium keratoplasticum* can be considered a representative fungi for fungal keratitis based on the common appearance of the *Fusarium solani* complex in MK cases [10], this organism was tested at longer time points. Thus, MPSs and lenses were challenged with *F. keratoplasticum* at the manufacturer’s disinfection time (Figure 2A), 24 h (Figure 2B), and 7 days (Figure 2C). Similar to Figure 1, the OPTI-FREE products (Puremoist, Express, and Replenish) and RevitaLens demonstrated the greatest log reduction at any time point, of at least 4.4 log reduction when disinfection was performed without lenses. Representative MPSs of each major biocide or biocide combination were chosen to move forward with the 24-h and 7-day time points: OPTI-FREE Puremoist (polyquaternium-1 and myristamidopropyl dimethylamine), RevitaLens (polyquaternium-1 and alexidine dihydrochloride), Lite (polyhexanide), and Biotrue (polyaminopropyl biguanide). RevitaLens and Biotrue achieved at least 4.0 log reduction by the 24-h time point (Figure 2B), but not at the manufacturer’s disinfection time (Figure 2A). Similar to the results from *S. marcescens*, Biotrue was again the only MPS to achieve greater disinfection efficacy with lenses than without (Figure 2A). OPTI-FREE Puremoist was the only MPS that produced a log reduction of 4.0 or greater among all lenses tested at both the disinfection time and 24-h time points (Figure 2A,B). However, by the 7-day time points, all MPSs, except for those containing polyhexanide or polyhexamethylene biguanide (such as Lite), achieved the 4.0 or greater log reduction disinfection efficacy among all lenses tested (Figure 2C). When examining product families at the manufacturer’s disinfection time, the OPTI-FREE products/Air Optix HG and RevitaLens/Vita outperformed the other product pairings. Further, renu Advanced/Ultra maintained a significantly greater disinfection efficacy than renu MultiPlus/Ultra, Lite/Vitality, Biotrue/Ultra, or Complete/Vita (*p* < 0.05). Biotrue/Ultra maintained greater efficacy than Lite/Vitality or Complete/Vita. Thus, even within product families, such as within a single manufacturer, there can be significant differences in disinfection efficacy at the manufacturer’s stated disinfection time, depending on which MPS is paired with the contact lens. This is again true when observing that RevitaLens/Vita performs significantly better than Complete/Vita, although it should be noted that the biocidal composition as well as the disinfection efficacy performance of these two MPSs are very different (Table 1) (*p* < 0.05). The OPTI-FREE products (Puremoist, Express, and Replenish) also maintained significantly higher disinfection efficacy at the disinfection time when paired with other manufacturer lenses as compared with that manufacturer lens’ own MPS (*p* < 0.05). Specifically, the OPTI-FREE products outperformed renu Advanced, renu MultiPlus, Lite, Biotrue, and Complete in this fashion (Figure 2A). Predictably, the 24-h and 7-day time periods possessed fewer of these differences as MPSs continued to disinfect over time. However, even at these time points, some product families were less effective than other manufacturers. Notably, at both the 24-h and 7-day time points, Lite/Vitality maintained significantly lower disinfection efficacy than OPTI-FREE Puremoist/Air Optix HG, RevitaLens/Vita, or Biotrue/Ultra. OPTI-FREE Puremoist/Vitality also maintained significantly greater disinfection efficacy than Lite/Vitality at both of these time points (Figure 2B,C) (*p* < 0.05). 

To further explore other *Fusarium* species, MPSs and their contact lenses were also challenged with *Fusarium chlamydosporum* AMC 5663 (Figure 3A), a clinical keratitis isolate of *Fusarium* AMC 1620 (Figure 3B), and *Fusarium roseum* AMC 5662 (see Figure S2, Supplemental Digital Content, which illustrates the quantification of the log reduction of *Fusarium roseum* at DT) for the manufacturer’s stated disinfection time. Challenges using *Fusarium chlamydosporum* produced the more typical differentiation between no-lens and with-lens disinfection efficacies among most products, with Biotrue being the exception and possessing no significant difference between lenses within the MPS (Figure 3A). This pathogen also again identified significant differences between and within product families. RevitaLens/Vita and Lite/Vitality demonstrated significantly lower disinfection efficacy than OPTI-FREE Puremoist or OPTI-FREE Express with the Air Optix HG lens (*p* < 0.05). When comparing OPTI-FREE products with other lenses, OPTI-FREE Puremoist and OPTI-FREE Express with Vita or with Ultra maintained significantly greater disinfection efficacy than RevitaLens/Vita or Lite/Ultra, respectively (*p* < 0.05). Biotrue/Ultra was also significantly less effective than OPTI-FREE Express/Ultra (*p* < 0.05). Finally, challenges using a clinical keratitis isolate of *Fusarium* AMC 1620 produced some of the lowest disinfection efficacies among all experiments conducted (Figure 3B). This isolate also uniquely resulted in lower no-lens disinfection efficacies compared to with-lens trials, particularly in OPTI-FREE Puremoist, OPTI-FREE Express, RevitaLens, and Biotrue. All of the OPTI-FREE products with the Air Optix HG lens possessed significantly higher disinfection efficacies than RevitaLens/Vita, renu MultiPlus/Ultra, Lite/Vitality, and Biotrue/Ultra (*p* < 0.05). The OPTI-FREE MPSs combined with Vita, Ultra, or Vitality also produced significantly greater disinfection efficacies than their original manufacturer products (RevitaLens, renu MultiPlus, Biotrue, or Lite, respectively) (*p* < 0.05). 

## 4. Discussion

Previous microbial keratitis (MK) outbreaks in the United States [1,2,3] and abroad [4,5,6] have indicated the need for abundant testing and disinfection regulation regarding contact lens products on the market. These infections are serious, potentially leading to corneal damage and blindness [1,3]. Importantly, these infections are linked to ineffective or misuse of contact lenses and lens care products [1]. Therefore, it is critical to understand differences in the disinfection efficacy of each product, and in combination with, contact lenses as each product would be in a real-world situation. This may enable consumers and physicians to have the most accurate information available for their contact lens product choices. Further, as biocide uptake by contact lens cases can introduce significant reductions to the presence and activity of MPSs [13,18], we aimed to incorporate testing of lenses in their manufacturer-provided cases. Thus, the goal of this study was to use industry standards in testing (International Standards Organization protocols 14729 and 18259) to examine nine different contact lens multipurpose solutions (MPSs), four different contact lenses, and four different common ocular pathogens at several time points. 

The wide range of biocides in preserved contact lens disinfection systems are notable and likely the root of many of the differences in disinfection efficacy. For instance, the OPTI-FREE MPS products (Puremoist, Express, and Replenish) use 0.001% of polyquaternium-1 and 0.0005–0.0006% of myristamidopropyl dimethylamine, and are the only products to use this combination or this amount of polyquaternium-1. Acuvue RevitaLens, renu Advanced, and Biotrue all also contain polyquaternium, although only 0.0001–0.0003%. Polyhexamethylene biguanide and polyaminopropyl biguanide (in quantities of 0.00005–0.0001%) are popular biocides and present in renu Advanced, renu MultiPlus, Biotrue, and Complete Easy Rub. Alexidine dihydrochloride (0.00016–0.0002%) is used in both renu Advanced and Acuvue RevitaLens. Two of the MPSs studied here rely solely on a single biocide, such as Lite (polyhexanide, 0.0001%) and Complete Easy Rub (polyhexamethylene biguanide, 0.0001%). Other components included in MPSs, such as the manufacturer’s cleaning, wetting, and comfort agents, may additionally impact the disinfection efficacy of the included biocides. This could account for differences in efficacy across products despite similar biocidal composition.

The results of this investigation also indicate a difference in disinfection efficacy based on the contact lenses themselves, due to differences noted between lenses even when using the same MPS. Each of the lenses here is made from a different material: Air Optix HG is made from lotrafilcon B, Acuvue Vita from senofilcon C, Ultra from samfilcon A, and Avaira Vitality from fanfilcon A. There is limited available information regarding the inherent inhibition of pathogenic colonization provided by popular contact lens materials, but the OPTI-FREE products have previously been shown to be superior in reducing bacterial colonization on a lotrafilcon A lens [19]. Conversely, lenses made with senofilcon A have been shown to possess higher bacterial loads than competitive lenses in the same conditions [20]. However, the current study, combined with previous investigations [13], provides valuable real-world information regarding the disinfection efficacy of popular contact lenses when used with popular MPSs. Importantly, differences between lenses were most evident in underperforming MPSs. That is, when the disinfection efficacy of the MPS was lower than those from other manufacturers, potential differences between lenses became more evident. 

Notably, the results of these experiments indicate that some lens–MPS combinations lose a significant amount of disinfection efficacy over no-lens or ISO 14729 disinfection trials. Among all lens–MPS combinations and pathogens, the OPTI-FREE products consistently demonstrate the highest amount of disinfection efficacy at the manufacturer’s disinfection time versus products from other manufacturers. RevitaLens, with all lens combinations, also outperformed in disinfection efficacy compared with other manufacturer products when challenged with *Fusarium keratoplasticum* ATCC 36031. Conversely, both Lite and Complete, with all lens combinations, underperformed compared with other-manufacturer products within all pathogens examined. Although some product families were largely internally consistent in terms of performance when their own highly similar products are paired together, there was a large amount of variation in Bausch + Lomb products. renu Advanced paired with Ultra performed consistently better than renu MultiPlus or Biotrue paired with Ultra.

It is also important to note how these results compare to industry standards for contact lens-related disinfection efficacy. ISO 18259 provides CLC manufacturers with the guidance to evaluate the compatibility of solutions used for disinfection with contact lenses and their cases using an antimicrobial endpoint method. The ISO 14729 stand-alone test maintains a disinfection primary criterion of a mean microorganism reduction of at least 99.9% (3.0 log reduction) by the manufacturer’s DT when challenged with bacteria. For yeast and mold, a mean reduction of at least 90% (1.0 log reduction) is required by DT, with no loss in efficacy at 24 h. Although ISO 18259 has no criteria, if ISO 14729 requirements are applied to the ISO 18259 performed in this study, OPTI-FREE Puremoist, RevitaLens, renu Advanced, and Biotrue all met the three log reduction criteria when combined with all tested lenses by DT when challenged with *Serratia marcescens*. Lite and Complete were the only MPSs not to meet the primary criteria of one log reduction for *Fusarium keratoplasticum* by DT for all lenses tested. RevitaLens and the OPTI-FREE products exceeded this criteria and achieved three log reduction by DT for all lenses tested after challenge with *F. keratoplasticum*. *Fusarium* did not regrow following disinfection, and all MPSs tested also did not lose efficacy at 24 h compared with DT. Finally, although all MPSs with all lenses tested achieved the one log reduction for *F. chlamydosporum*, only the OPTI-FREE products achieved this criteria when tested against the clinical keratitis isolate of *Fusarium* AMC 1620.

In conclusion, the OPTI-FREE products were the only products to consistently perform significantly better than other products within all microorganisms tested, which was true for all no-lens and with-lens combinations. RevitaLens and renu Advanced also performed significantly better than renu MultiPlus, Lite, Biotrue, and Complete when challenged with *S. marcescens* and *F. keratoplasticum*. These products consistently met ISO 14729 primary criteria as applied to ISO 18259 testing methodology for contact lens products, whereas the products that failed to meet ISO 14729 primary criteria for ISO 18259 demonstrated a significant loss in antimicrobial efficacy due to the presence of contact lenses. As MPS products are never used in the absence of contact lenses, the assessment of MPS antimicrobial efficacy in the presence of contact lenses and lens cases is critical to gain a true understanding of an MPS’s performance in the hands of a contact lens patient. 

## Figures and Tables

**Table 1:** Multipurpose solutions (and their manufacturers, biocides, and stated disinfection times) and contact lenses used.

Manufacturer	Product	Composition	Details
Alcon^®^ Fort Worth, TX, USA	AIR OPTIX^®^ plus HydraGlyde^®^	lotrafilcon B	Group V
	OPTI-FREE^®^ Puremoist^®^	polyquaternium-1 (0.001%), myristamidopropyl dimethylamine (0.0006%)	6 h DT
	OPTI-FREE^®^ Express^®^	polyquaternium-1 (0.001%), myristamidopropyl dimethylamine (0.0005%)	6 h DT
	OPTI-FREE^®^ Replenish^®^	polyquaternium-1 (0.001%), myristamidopropyl dimethylamine (0.0005%)	6 h DT
Johnson & Johnson New Brunswick, NJ, USA	ACUVUE^®^ VITA^®^	senofilcon C	Group V
	Complete^®^ Easy Rub	polyhexamethylene biguanide (0.0001%)	6 h DT
	ACUVUE^®^ RevitaLens^®^	polyquaternium-1 (0.0003%), alexidine dihydrochloride (0.00016%)	6 h DT
Bausch + Lomb^®^ Rochester, NY, USA	ULTRA^®^	samfilcon A	Group V
	renu^®^ Advanced Formula	polyquaternium (0.00015%), alexidine dihydrochloride (0.0002%), polyaminopropyl biguanide (0.00005%)	4 h DT
	renu^®^ MultiPlus	polyaminopropyl biguanide (0.0001%)	4 h DT
	Biotrue^®^	polyaminopropyl biguanide (0.00013%), polyquaternium (0.0001%)	4 h DT
CooperVision^®^ Lake Forest, CA, USA	Avaira Vitality™	fanfilcon A	Group V
	Lite™	polyhexanide (0.0001%)	6 h DT

## Data Availability

Data is contained within the article and supplementary material.

## Supplementary Materials

The following are available online at https://www.mdpi.com/article/10.3390/microorganisms9102173/s1, Table S1: Inoculum Control CFU/mL of each microorganism, Figure S1: Log reduction of *Serratia marcescens* at 24 h, and Figure S2: Log reduction of *Fusarium roseum* at DT.
