# Supplementary material for: Antimicrobial Efficacy of Contact Lens Solutions Assessed by ISO Standards
Source: Microorganisms. 2021 Oct 19;9(10):2173. doi: 10.3390/microorganisms9102173 (PMC8540466; doi:10.3390/microorganisms9102173)
Supplement: Supplementary file 1 [file microorganisms-09-02173-s001.zip › McAnally AEEMC Supplemental.pdf]

Supplemental Digital Content:

Table S1. Each microorganism with the testing condition and its inoculum control CFU/mL.

Pathogen	CFU/mL
Fusarium chlamydosporum AMC 5663	1.6 - 8.5 x 10^5
Fusarium keratoplasticum ATCC 36031	2.2 - 3.1 x 10^5
Fusarium roseum AMC 5662	1.1 – 2.2 x 10^5
Clinical keratitis isolate *Fusarium spp.* AMC 1620	1.5 - 4.0 x 10^5
Serratia marcescens ATCC 36031	2.9 – 9.6 x 10^5

Figure S1. Multi-purpose solutions (MPSs) and contact lenses challenged with *Serratia marcescens* ATCC 13880 at the manufacturer's disinfection time (DT). Disinfection efficacy is stated in mean ± standard error log reduction compared to inoculum control. * $p < 0.05$ vs. No Lens challenge at the same time point and within the same MPS. n=3/group.

Figure S2. Multi-purpose solutions (MPSs) and contact lenses challenged with *Fusarium roseum* AMC 5662 at the manufacturer's disinfection time (DT). Disinfection efficacy is stated in mean ± standard error log reduction compared to inoculum control. * $p < 0.05$ vs. No Lens challenge at the same time point and within the same MPS. Statistical differences between product families: *a* $p < 0.05$ vs. OPTI-FREE (OF) Puremoist with Air Optix HG, *b* $p < 0.05$ vs. OPTI-FREE Express with Air Optix HG, *c* $p < 0.05$ vs. OPTI-FREE Replenish with Air Optix HG; *aa* $p < 0.05$ vs. OPTI-FREE Puremoist with other manufacturer lens, *bb* $p < 0.05$ vs. OPTI-FREE Express with other manufacturer lens, *cc* $p < 0.05$ vs. OPTI-FREE Replenish with other manufacturer lens. n=3/group.
