# Supplementary figures and images for: Antimicrobial Efficacy of Contact Lens Solutions Assessed by ISO Standards
Source: Microorganisms. 2021 Oct 19;9(10):2173. doi: 10.3390/microorganisms9102173 (PMC8540466; doi:10.3390/microorganisms9102173)

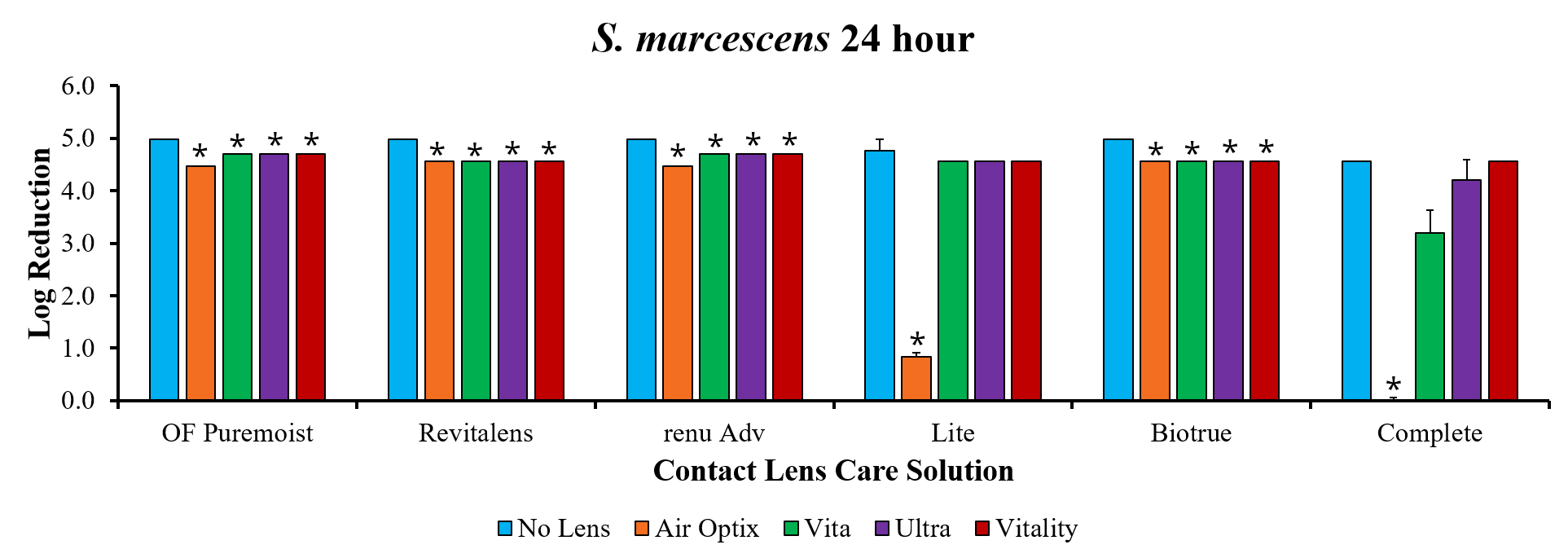

Supplement: Supplementary file 1 [file microorganisms-09-02173-s001.zip › McAnally AEEMC Figure S1.tif]

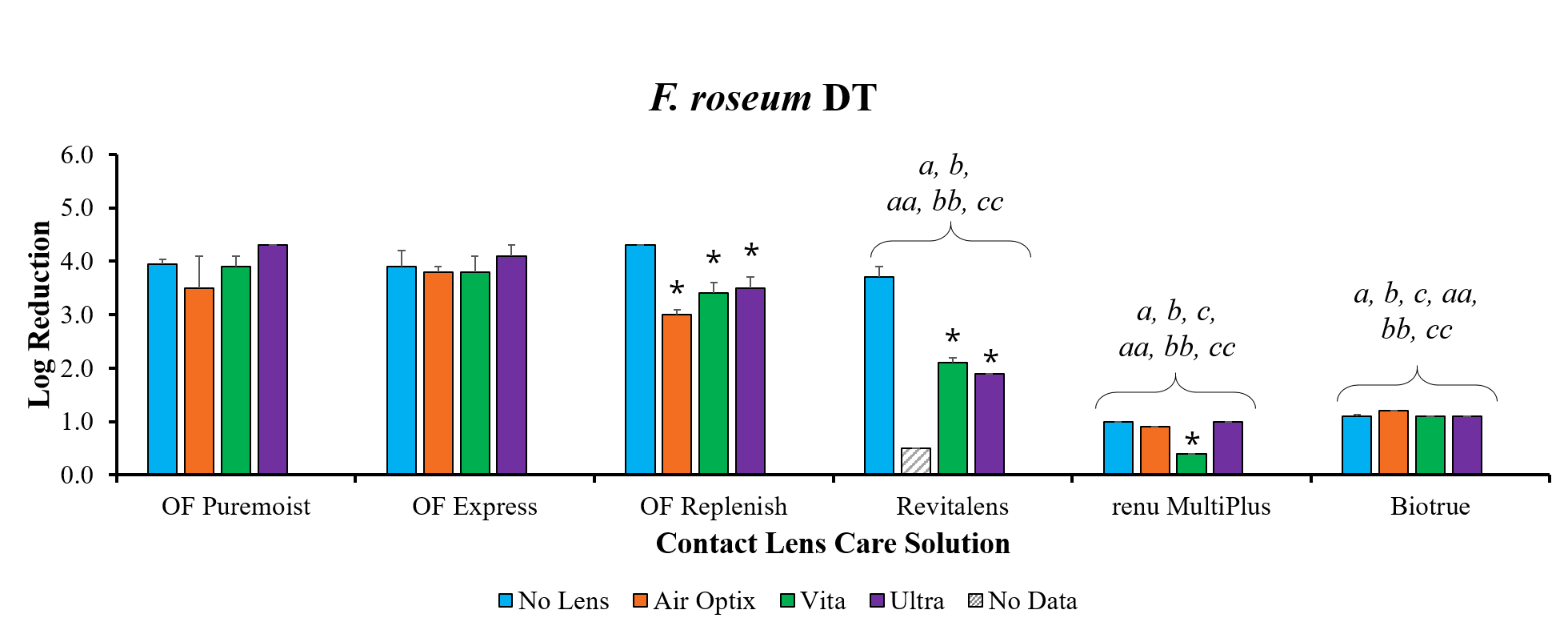

Supplement: Supplementary file 1 [file microorganisms-09-02173-s001.zip › McAnally AEEMC Figure S2.tif]
